# Prognostic Value of the New Prostate Cancer International Society of Urological Pathology Grade Groups

**DOI:** 10.3389/fmed.2017.00157

**Published:** 2017-09-29

**Authors:** Anne Offermann, Silke Hohensteiner, Christiane Kuempers, Julika Ribbat-Idel, Felix Schneider, Finn Becker, Marie Christine Hupe, Stefan Duensing, Axel S. Merseburger, Jutta Kirfel, Markus Reischl, Verena Lubczyk, Rainer Kuefer, Sven Perner

**Affiliations:** ^1^Pathology of the University Hospital Schleswig-Holstein, Campus Luebeck and Research Center Borstel, Leibniz Center for Medicine and Biosciences, Luebeck, Germany; ^2^Department of Pathology, Klinik am Eichert Alb Fils Kliniken, Goeppingen, Germany; ^3^Department of Urology, Klinik am Eichert Alb Fils Kliniken, Goeppingen, Germany; ^4^Department of Urology, University Hospital Schleswig-Holstein, Luebeck, Germany; ^5^Department of Urology, Section of Molecular Urooncology, University Hospital Heidelberg, Heidelberg, Germany; ^6^Institute of Pathology and Center for Integrated Oncology Cologne Bonn, University Hospital of Bonn, Bonn, Germany; ^7^Institute for Applied Computer Science, Karlsruhe Institute of Technology, Karlsruhe, Germany

**Keywords:** prostate cancer, Gleason score, International Society of Urological Pathology Grade Groups, prognostic biomarker, cancer grading

## Abstract

Gleason grading is the best independent predictor for prostate cancer (PCa) progression. Recently, a new PCa grading system has been introduced by the International Society of Urological Pathology (ISUP) and is recommended by the World Health Organization (WHO). Following studies observed more accurate and simplified grade stratification of the new system. Aim of this study was to compare the prognostic value of the new grade groups compared to the former Gleason Grading and to determine whether re-definition of Gleason Pattern 4 might reduce upgrading from prostate biopsy to radical prostatectomy (RP) specimen. A cohort of men undergoing RP from 2002 to 2015 at the Hospital of Goeppingen (Goeppingen, Germany) was used for this study. In total, 339 pre-operative prostatic biopsies and corresponding RP specimens, as well as additional 203 RP specimens were re-reviewed for Grade Groups according to the ISUP. Biochemical recurrence-free survival (BFS) after surgery was used as endpoint to analyze prognostic significance. Other clinicopathological data included TNM-stage and pre-operative PSA level. Kaplan–Meier analysis revealed risk stratification of patients based on both former Gleason Grading and ISUP Grade Groups, and was statistically significant using the log-rank test (*p* < 0.001). Both grading systems significantly correlated with TNM-stage and pre-operative PSA level (*p* < 0.001). Higher tumor grade in RP specimen compared to corresponding pre-operative biopsy was observed in 44 and 34.5% of cases considering former Gleason Grading and ISUP Grade Groups, respectively. Both, former Gleason Grading and ISUP Grade Groups predict survival when applied on tumors in prostatic biopsies as well as RP specimens. This is the first validation study on a large representative German community-based cohort to compare the former Gleason Grading with the recently introduced ISUP Grade Groups. Our data indicate that the ISUP Grade Groups do not improve predictive value of PCa grading and might be less sensitive in deciphering tumors with 3 + 4 and 4 + 3 pattern on RP specimen. However, the Grade Group system results less frequently in an upgrading from biopsy to the corresponding RP specimens, indicating a lower risk to miss potentially aggressive tumors not represented on biopsies.

## Introduction

Prostate cancer (PCa) is the most common cancer type among men worldwide accounting for more than 20% of all newly diagnosed cancers (1). Patients show a highly variable course of disease, resulting in a major challenge for clinical management (2). Consequently, it is of utmost importance to stratify patients with early-stage disease into certain risk groups, predicting the probability of remaining an indolent or progressing to an aggressive form of PCa. In addition to clinical stage and serum PSA level, Gleason grading of the tumor is a powerful prognostic marker at time point of diagnosis and has major impact on therapy decision (3). A limitation of Gleason grading is upgrading from biopsy to radical prostatectomy (RP) specimens, most often in lower grade tumors, which is associated with worse outcome of patients (4). In addition, considerable interobserver variability limits PCa grading to be reproducible in a subset of cases (5).

Since its introduction (6), the Gleason Grading system has undergone several revisions in order to improve reproducibility and prognostic value (7). Most recently, a new grading system has been proposed by the International Society of Urological Pathology (ISUP) (8) and is integrated into the 2016 edition of the WHO classification of Tumor of the Urinary System and Male Genital Organs (9). Major modifications of the ISUP Grade Groups toward the last update in 2010 include defining five distinct Grade Groups based on the Gleason score: Grade Group 1 = Gleason score ≤6, Grade Group 2 = Gleason score 3 + 4 = 7, Grade Group 3 = Gleason score 4 + 3 = 7, Grade Group 4 = Gleason score 8, Grade Group 5 = Gleason scores ≥9, as well as modified morphological criteria for Gleason pattern 4 (9). Consequently, various growth patterns, including ill-formed, fused, cribriform, and glomeruloid glands are considered as Gleason grade 4 tumors. In consideration of a multi-institutional validation study (10), the ISUP Grade Groups emerged as more accurate and simplified classification to stratify tumors than the current system (9).

A number of recently published studies independently validated the ISUP Grade Groups as prognostic marker for biochemical recurrence (BR) as well as disease-specific death of patients (11–14). While some observed prognostic benefits of the new classification, others reported no significant difference to the former Gleason Grading (12, 15). It has to be considered that the mentioned studies vary regarding end points for survival analysis and treatment strategies of patients, resulting in limited comparability.

The aim of our study was to compare the prognostic value as well as the frequency of upgrading from diagnostic biopsy to RP specimen between the former Gleason Grading and the ISUP Grade Groups. Diagnostic biopsies and RP specimens were re-reviewed and graded according to the new classification.

## Materials and Methods

A cohort including 339 prostatic pre-operative biopsies and corresponding RP specimens, as well as additional 203 RP specimens from patients who were treated between 2002 and 2015 at the Hospital of Goeppingen (Goeppingen, Germany) was used for the present study. Only biopsies from patients who achieved subsequent RP were included for survival analyses. All RP specimens and biopsies were initially graded by pathologists of the Hospital of Goeppingen, and afterward re-graded in a centralized manner by an expert GU pathologist according to the current ISUP grading system. BR was defined as postoperative PSA increase of ≥0.2 ng/ml. Patients’ characteristics are summarized in Table 1.

**Table 1:** Baseline characteristics of patients.

Median (range) preoperative PSA (ng/ml)	7.35 (0.0–3202.0)
**Preoperative PSA**	
<10 ng/ml [*n* (%)]	383 (70.9)
>10 ng/ml [*n* (%)]	157 (29.1)
**Pathological T stage [*n* (%)]**	
pT2	348 (67.8)
pT3a	82 (16.0)
pT3b	80 (15.6)
pT4	3 (0.6)
Extraprostatic tumor expansion [*n* (%)]	165 (32.2)
**Lymph node status [*n* (%)]**	
N0	492 (90.8)
N1	50 (9.2)
**Surgical margin [*n* (%)]**	
pR0	413 (76.2)
pR1	129 (23.8)
**Former Gleason Score [*n* (%)] at radical prostatectomy (RP), *n* = 542**	
≤6	173 (31.9)
3 + 4	207 (38.2)
4 + 3	50 (9.2)
8	72 (13.3)
≥9	40 (7.4)
**International Society of Urological Pathology (ISUP) Grade Group [*n* (%)] at RP, *n* = 542**	
1	170 (31.4)
2	216 (39.9)
3	82 (15.1)
4	42 (7.7)
5	32 (5.9)
**Former Gleason Score [*n* (%)] at diagnostic biopsy *n* = 339**	
≤6	224 (66.1)
3 + 4	87 (25.7)
4 + 3	12 (3.5)
8	12 (3.5)
≥9	4 (1.2)
**ISUP Grade Group [*n* (%)] at diagnostic biopsy *n* = 339**	
1	187 (55.2)
2	66 (19.5)
3	29 (8.6)
4	34 (10.0)
5	23 (6.8)

Ethical approval for using human material in this study was obtained from the Internal Review Board of the University Hospital of Bonn (264/11). The study participants were anonymized before their specimens were included to this retrospective study cohort.

Chi-square tests were used for comparison of BR between the former Gleason Grading and the ISUP Grade Groups as categorical variables. To analyze univariable differences in BFS between the former Gleason Grading and the ISUP Grade Groups, the log-rank test was used. Kaplan–Meier curves illustrate BFS after treatment stratified by the former Gleason Grading and the ISUP Grade Groups. Multivariable Cox proportional hazards models were performed to identify independent prognostic factors. Grading on biopsies were adjusted for the log of pre-operative PSA (≤/> 10 ng/ml), and grading on RP specimens were adjusted for the log of pre-operative PSA (≤/> 10 ng/ml), pathological T-stage (pT2, pT3a, pT3b, pT4), lymph node status (pN0, pN1), and surgical margin status (pR0, pR1). Gleason Score 3 + 3 and Grade Group 1 were used as reference group for hazard ratios.

All statistics were done with SPSS vers. 2.0 (Inc., Chicago, IL, USA).

Study design, patient characteristics, methods, and statistical analysis as well as data presentation and discussion have been performed according to the REMARK (REporting recommendations for tumor MARKer prognostic studies) guidelines (16).

## Results

Biochemical recurrence-free survival was used as end point to compare prognostic value of different grading systems. The frequency of BR at any time point after treatment increases with both rising former Gleason Grading and ISUP Grade Groups as assessed on RP specimens as well as diagnostic biopsies (Table 2). The rate of BR of tumors graded with 3 + 3 on RP (9.2%) as well as biopsies (21.4%) was higher compared to tumors graded with Grade Group 1 (5.9% at RP, 13.7% at biopsy). In RP specimens, we observed lower frequency of BR in Grade Group 4 tumors (42.9%) compared to Grade Group 3 tumors (53.7%), but clearly higher and lower rate compared to Grade Group 2 (23.1%) and Grade Group 5 (71.9) tumors, respectively (Table 2). Statistical analysis revealed significant association between both, the former Gleason Grading and the ISUP Grade Groups, and risk for BR (Chi-Square, *p* < 0.001) (Table 2).

**Table 2:** Association between different grading systems at RP or diagnostic biopsies and frequency of biochemical recurrence (BR).

	BR [*n* (%)]	*p*-Value
Former Gleason Score at radical prostatectomy (RP)		<*0.001*
≤6	16 (9.2)	
3 + 4	39 (18.8)	
4 + 3	23 (46.0)	
8	40 (55.6)	
≥9	27 (67.5)	
International Society of Urological Pathology (ISUP) Grade Group at RP		<*0.001*
1	10 (5.9)	
2	50 (23.1)	
3	44 (53.7)	
4	18 (42.9)	
5	23 (71.9)	
Former Gleason Score at diagnostic biopsy		<*0.001*
≤6	48 (21.4)	
3 + 4	31 (35.6)	
4 + 3	8 (66.7)	
8	8 (66.7)	
≥9	3 (75.0)	
ISUP Grade Group at diagnostic biopsy		<*0.001*
1	26 (13.9)	
2	23 (34.8)	
3	13 (44.8)	
4	18 (52.9)	
5	18 (78.3)	

**Figure 1:**
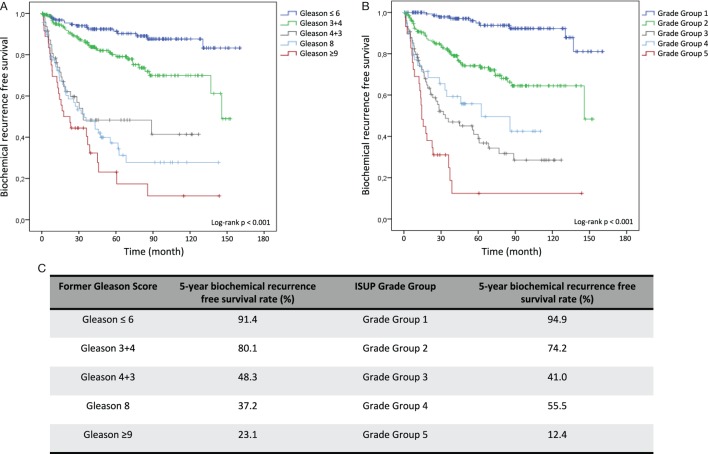
Kaplan–Meier analysis for biochemical recurrence free survival (BFS) according to the former Gleason Grading and International Society of Urological Pathology (ISUP) Grade Groups on RP specimens. **(A)** Biochemical recurrence-free survival (BFS) stratified by Gleason ≤6, Gleason 3 + 4, Gleason 4 + 3, Gleason 8 and Gleason ≥9. **(B)** BFS stratified by Grade Group 1, Grade Group 2, Grade Group 3, Grade Group 4, and Grade Group 5. **(C)** 5-year BFS rate in % for grading according to former Gleason Grading or ISUP Grade Group.

**Figure 2:**
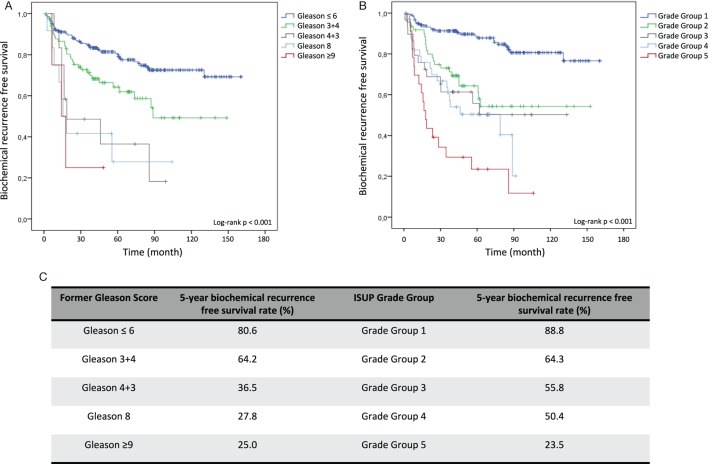
Kaplan–Meier analysis for biochemical recurrence free survival (BFS) according to the former Gleason Grading and International Society of Urological Pathology (ISUP) Grade Groups on diagnostic biopsies. **(A)** BFS stratified by Gleason ≤6, Gleason 3 + 4, Gleason 4 + 3, Gleason 8, and Gleason ≥9. **(B)** BFS stratified by Grade Group 1, Grade Group 2, Grade Group 3, Grade Group 4, and Grade Group 5. **(C)** 5-year BFS rate in% for grading according former Gleason Grading or ISUP Grade Group.

Kaplan–Meier curves illustrate reduced cumulative BFS time in tumors with higher former Gleason Grading and ISUP Grade Groups at both, RP and diagnostic biopsy, allowing significant risk stratification of patients over time (log-rank test < 0.001) (Figures 1A,B and 2A,B). Concordantly, higher former Gleason Grade and ISUP Grade Group associates with lower 5-year-BFS rates of patients (Figures 1C and 2C). The 5-year-BFS rate of tumors graded with former Gleason Score 3 + 3 on RP (91.4%) as well as biopsies (80.6%) was lower compared to tumors graded with ISUP Grade Group 1 (94.9% at RP, 88.8% at biopsy). By contrast, 5-year-BFS rate of ISUP Grade Group 2 (74.2%) and ISUP Grade Group 3 (41%) tumors at RP were higher compared to tumors graded with former Gleason Score 3 + 4 (48.3%) and 4 + 3 (37.2%). Kaplan–Meier curve shows higher cumulative BFS and 5-year BFS rate of patients with ISUP Grade Group 4 tumors compared to ISUP Grade Group 3 tumors (Figures 1B,C).

Using former Gleason Score 3 + 3 and ISUP Grade group 1 as reference group in multivariable Cox analysis, RP specimens with former Gleason Score (ISUP Grade group) 3 + 4 (2), 4 + 3 (3), 8 (4), >9 (5) are associated with 1.78 (4.08), 4.81 (10.25), 6.53 (7.81) and 6.57 (12.93) times increased hazard for BR, respectively (Table 3). When determined at diagnostic biopsies, former Gleason Score (ISUP Grade group) 3 + 4 (2), 4 + 3 (3), 8 (4), >9 (5) is associated with 2.07 (3.25), 3.55 (3.98), 4.55 (4.93), and 7.11 (9.40) times increased hazard for BR, respectively (Table 4). In multivariate analyses, RP specimens were adjusted to lymph node status, surgical margin status, T-stage, and pre-operative PSA (Table 3), and biopsies to pre-operative PSA (Table 4). In multivariate analysis, RP specimens graded as 3 + 4 by the former Gleason Grading is associated with 1.78 times increased hazard for BR referring to 3 + 3 tumors which is statistically not significant (0.058) (Table 3). Collectively, hazard ratios for BR are higher for tumors graded by the ISUP Grade Groups compared to the former Gleason Grading.

**Table 3:** Multivariate Cox analysis for biochemical recurrence free survival (BFS) according to different grading systems on RP specimens (HR = hazard ratio, CI = confidence interval, RP = radical prostatectomy).

Clinicopathological parameter	Multivariate analysis	
	HR (95% CI)	*p*-Value
**Former Gleason Score [*n* (%)] at radical prostatectomy (RP)**		
≤6		
3 + 4	1.78 (0.98–3.22)	*0.058*
4 + 3	4.81 (2.47–9.38)	<*0.001*
8	6.53 (3.53–12.05)	<*0.001*
≥9	6.57 (3.34–12.91)	<*0.001*
**International Society of Urological Pathology Grade Group [*n* (%)] at RP**		
1		
2	4.08 (2.04–8.17)	<*0.001*
3	10.25 (5.01–20.96)	<*0.001*
4	7.81 (3.51–17.36)	<*0.001*
5	12.93 (5.70–29.31)	<*0.001*

**Table 4:** Multivariate Cox analysis for Biochemical recurrence-free survival (BFS) according to different grading systems on biopsies (HR = hazard ratio, CI = confidence interval, RP = radical prostatectomy).

Clinicopathological parameter	Multivariate analysis	
	HR (95% CI)	*p*-Value
**Fomer Gleason Score [*n* (%)] at diagnostic biopsy**		
≤6		
3 + 4	2.07 (1.31–3.26)	*0.002*
4 + 3	3.55 (1.6–7.92)	*0.002*
8	4.55 (2.13–9.73)	<*0.001*
≥9	7.11 (2.19–23.13)	*0.001*
**International Society of Urological Pathology Grade Group [*n* (%)] at diagnostic biopsy**		
1		
2	3.25 (1.85–5.73)	<*0.001*
3	3.98 (2.00–7.9)	<*0.001*
4	4.93 (2.68–9.08)	<*0.001*
5	9.40 (5.08–17.41)	<*0.001*

Overall, in nine patients, disease recurrences appeared as local tumor recurrence or development of distant metastases (assigned as clinical recurrence), partially after diagnosis of BR. Only 6 patients died from PCa. Association between different grading systems and clinical recurrence and disease-specific death are listed in Table 2.

Both the former Gleason Grading and the ISUP Grade Groups significantly correlate with T-stage, lymph node status and pre-operative PSA level (Chi-square *p* < 0.001).

Upgrading from diagnostic biopsy to RP specimen occurred in 34.5% considering ISUP Grade Groups, and more often considering the former Gleason Grading (44.0%) (Table 3). The vast majority of cases affected low grade tumors independent of the grading system. Frequency and distribution of upgrading are listed in Table 5.

**Table 5:** Frequency and distribution of upgrading from diagnostic biopsy to RP.

Upgrading from biopsy to RP	*n* (%)	Upgrading from biopsy to RP	*n* (%)
Former Gleason Score (total)	149 (44.0)	International Society of Urological Pathology Grade Group (total)	117 (34.5)
≤6 → 3 + 4	75 (22.1)	1 → 2	74 (21.8)
≤6 → 4 + 3	13 (3.8)	1 → 3	12 (3.5)
≤6 → 4 + 4	18 (5.3)	1 → 4	5 (1.5)
≤6 → ≥ 9	8 (2.4)	1 → 5	1 (0.3)
3 + 4 → 4 + 3	8 (2.4)	2 → 3	10 (2.9)
3 + 4 → 4 + 4	9 (2.7)	2 → 4	3 (0.9)
3 + 4 → ≥ 9	7 (2.1)	2 → 5	2 (0.6)
4 + 3 → 4 + 4	2 (0.6)	3 → 4	4 (1.2)
4 + 3 → ≥ 9	3 (0.9)	3 → 5	2 (0.6)
4 + 4 → ≥ 9	6 (1.8)	4 → 5	4 (1.2)

## Discussion

Collectively, our findings confirm that the recently introduced ISUP Grade Groups independently predicts BR after treatment when conducted on both, RP specimens and diagnostic biopsies. Thus, these data support previous suggestions of the ISUP meeting 2014 to include ISUP Grade Groups into pathology reports (9).

Patients with low-risk disease are constantly risk stratified as tumors on diagnostic biopsies with ISUP Grade Group 2, 3, 4, and 5 exhibited a 3.25, 3.98, 4.93, and 9.40 hazard increased risk for BR compared to ISUP Grade Group 1 tumors, respectively (Table 4). Importantly, therapy decision is made based on the grading of diagnostic biopsies, PSA blood level, clinical tumor stage, and individual factors. With the objective to avoid over-treatment of indolent PCa, alternative regimes such as active surveillance and watchful waiting are increasingly applied worldwide (17). Among others, criteria for active surveillance include Gleason Score ≤6 in biopsy specimens to select patients with potential low-risk disease (18). Based on our results showing a 5-year BFS rate of 88.8% in ISUP Grade Group 1 biopsies, the recently introduced ISUP Grade Groups are sensitive markers to identify low-risk patients who should not undergo radical treatment approaches. In our evaluation, the ISUP Grade Groups emerged as even more sensitive compared to the former Gleason Grading (5-year BFS rate of 80.6% in former Gleason Score 3 + 3 biopsies) (Figure 2C).

Various growth patterns, including ill-formed, fused, cribriform, and glomeruloid glands, are considered as Gleason grade 4 tumors in the ISUP Grade Groups (9). In general, diagnosis of Gleason pattern 4 is associated with the highest interobserver variability among pathologists. Evaluating the interobserver reproducibility of individual Gleason pattern 4, a recently published study concluded consensus on cribriform and glomeruloid patterns but lacking consensus on ill-formed and fused glands (18). While our re-evaluation of RP specimens significantly differentiates ISUP Grade Group 1, ISUP Grade Group 2, and ISUP Grade Group 3 tumors exhibiting BR rates of 5.9, 23.1, and 53.7%, respectively, results show reduced BR frequency of ISUP Grade Group 4 tumors (42.9%) (Table 2) and higher cumulative BFS (Figures 1B,C) compared to ISUP Grade Group 3 tumors. This observation might result from various factors, including modified definition of Gleason pattern 4, partially suggesting to constitute a borderline pattern with reduced interobserver reproducibility (19). In addition, the proportion of ISUP Grade Group 4 tumors was relatively low accounting for 7.7% (42/542) of all RP specimens, resulting in limited informative value. Finally, general interobserver variability might have contributed to this discrepancy. Interestingly, a comparable observation has been made by Loeb et al (12), reporting a 4-year BFS rate of 77% for ISUP Grade Group 4 on biopsy, and a slightly lower rate for ISUP Grade Group 3 tumors (74%). Obviously, this result derives from grading on biopsies associated with upgrading to RP, and the discrepancy is marginal. However, overall data give evidence that separation Gleason pattern 3 from pattern 4 and reporting its proportion remains challenging.

Multiple studies revealed upgrading from diagnostic biopsy to corresponding RP specimen reporting incidences between 14 and 51% with a mean of 36% (20). Major issues emerged during recent years as Gleason grading on diagnostic biopsy has significant impact on therapy decision at time point of diagnosis to select patients for surgery, radiation or active surveillance. Upgrading indicates potential undertreatment of patients with assumed low-risk disease and is associated with worse outcome (21). Several updates of the Gleason grading system such as reporting the most common and highest Gleason pattern on biopsy, re-defining pattern 4 as well as increasing the number of biopsies have been performed in order to improve its prediction accuracy and reduce upgrading (20). We observed upgrading from biopsy to corresponding RP specimen in 44.0% of cases considering the former Gleason Grading, and less common after re-grading according to the ISUP Grade Groups (34.5%) (Table 5). In accordance with published data, the vast majority of upgrading occurred from tumors with former Gleason Score ≤6 and ISUP Grade Group 1, most often to former Gleason Score 3 + 4 and ISUP Grade Group 2 at RP (Table 5). Equal observation has been reported by a recently published study showing most frequent upgrades from biopsy ISUP Grade Group 1 to RP ISUP Grade Group 2 (22). Collectively, our results give evidence that the modified definition of pattern 4 reduces upgrading, thus associates with improved predictive accuracy and lower risk of undertreatment.

Since patients with highly aggressive disease are underrepresented in our study, correlation between grading and important endpoints such as development of metastasis and PCa specific death is limited. As discussed above, ISUP Grade Group 4 was diagnosed in 7.7% of all RP, limiting statistical significance and informative value within this group. Furthermore, former Gleason Grading of biopsies and RP specimens was performed by pathologists from different institutes which might influence higher incidence of upgrading.

To conclude, our data support the previously accepted ISUP Grade Groups according to the ISUP meeting 2014 as independent prognostic marker for PCa. Diagnosis of Gleason pattern 4 and distinguishing Grade Group 3 from Grade Group 4 remains challenging, considering limitations of this study and general interobserver variability. On RP specimens, we did not observe prognostic benefits of the ISUP Grade Groups compared to the former Gleason Grading. However, in our study the incidence of upgrading from biopsy to corresponding RP was lower by using the ISUP Grade Groups, giving evidence that the ISUP Grade Groups might improve predictive accuracy as assigned on diagnostic biopsies.

## Ethics Statement

Ethical approval for using human material in the present study was obtained from the Internal Review Board of the University Hospital of Bonn (264/11). The study participants were anonymized before their specimens were included to this retrospective study cohort.

## Author Contributions

SP, RK, VL, and AO designed the study approach. SH, FS, CK, JR-I, FB, and AO performed microscopic and histolpathologic investigation. MR, AO, and MH performed statistics. SP, RK, VL, AM, SD, JK, AO, and MH interpreted data. SP and AO wrote the manuscript. All authors reviewed and approved the final manuscript.

## Conflict of Interest Statement

The authors declare that the research was conducted in the absence of any commercial or financial relationships that could be construed as a potential conflict of interest.
